# Wood’s Medical and Surgical Monographs

**Published:** 1889-10

**Authors:** 


**Wood's Medical and Surgical Monographs.** Volume III., No. 3, September, 1889. 8vo, pp. 288. New York: William Wood & Co., 56 and 58 Lafayette place. Price, single number, $1.00, or $10.00 a year.

The ninth number of this most valuable series of medical publications comes promptly to hand, and is every way equal to the preceding ones in interest and importance. The titles in the current number are: Congestive Neurasthenia and Nerve Depression, by E. G. Whittle, M. D.; The Art of Embalming, by Benj. Ward Richardson,

M. D.; The Etiology, Diagnosis, and Treatment of Tuberculosis, by Dr. H. Von Ziemssen; Psycho-Therapeutics, or Treatment by Hypnotism, by Dr. C. Lloyd Tuckey; Sexual Activity and the Critical Period in Man and Woman, by Dr. Louis De Sèrê.

The index to Volume III. accompanies this book. If any physician has not already subscribed to the series, he should make haste to do so, for it is a rare opportunity to obtain useful literature at a marvelously low price. At all events, he should secure the September number.

